# Synthesis of Unsaturated Polyester Resins from Various Bio-Derived Platform Molecules

**DOI:** 10.3390/ijms160714912

**Published:** 2015-07-02

**Authors:** Thomas J. Farmer, Rachael L. Castle, James H. Clark, Duncan J. Macquarrie

**Affiliations:** Green Chemistry Centre of Excellence, Department of Chemistry, University of York, Heslington, York YO10 5DD, UK; E-Mails: thomas.farmer@york.ac.uk (T.J.F.); rc566@york.ac.uk (R.L.C.); duncan.macquarrie@york.ac.uk (D.J.M.)

**Keywords:** polymerisation, green chemistry, michael addition, polymers, renewable resources, transesterification, bio-derived platform molecules, itaconates

## Abstract

Utilisation of bio-derived platform molecules in polymer synthesis has advantages which are, broadly, twofold; to digress from crude oil dependence of the polymer industry and secondly to reduce the environmental impact of the polymer synthesis through the inherent functionality of the bio-derived platform molecules. Bulk polymerisation of bio-derived unsaturated di-acids has been employed to produce unsaturated polyester (UPEs) which have been analysed by GPC, TGA, DSC and NMR spectroscopy, advancing on the analysis previously reported. UPEs from the diesters of itaconic, succinic, and fumaric acids were successfully synthesised with various diols and polyols to afford resins of *M*_N_ 480–477,000 and *T*_g_ of −30.1 to −16.6 °C with solubilities differing based on starting monomers. This range of properties allows for many applications and importantly due to the surviving Michael acceptor moieties, solubility and cross-linking can be specifically tailored, post polymerisation, to the desired function. An improved synthesis of itaconate and succinate co-polymers, *via* the initial formation of an itaconate *bis*-diol, is also demonstrated for the first time, resulting in significantly improved itaconate incorporation.

## 1. Introduction

Unsaturated polyesters (UPEs) are a large family of polymers with a wide range of applications stemming from their ability to undergo various post-polymerisation reactions. They can be utilised in high-gloss coatings [1], insulating materials [1], drug delivery systems [2] and biomedical applications such as tissue engineering [2]. The polymer industry currently relies heavily on petroleum-based feedstocks [3] but with fossil fuel resources due to be soon depleted [4], biomass sources are of increasing interest in this research area [3]. Bio-derived di-acids are obvious choices due to their potential for incorporation into polyesters and their increasing production from carbohydrates via fermentation [5,6,7]. Environmentally damaging oxidation steps from the current petrochemical route to polyester monomers can also be avoided because of the existing oxygenation in the bio-based alternatives [7]. Additionally, some bio-derived UPEs contain inherent bio-degradability [8] and biocompatibility [2].

The synthesis of bio-derived UPEs is already well documented in the literature. Monomers/oligomers based on itaconic acid (IA) are frequently utilised due to the *exo*-double bond functionality of this io-derived di-acid [9,10,11,12,13]. Because of its conjugation with the adjacent carbonyl, the vinyl group acts as a Michael acceptor and allows for post-polymerisation functionalisation and hence property tuning of the itaconate polyesters [7,14,15,16,17]. A wide variety of compounds have been incorporated into the oligomers alongside the itaconate moiety such as; alcohols (e.g., 1,4-butanediol [10], ethylene glycol [10], trimethylolpropane [9], sorbitol [9], isosorbide [18] or isoamyl alcohol [12]); epoxides [19] (e.g., propylene oxide, glycidyl ether, styrene oxide or epichlorohydrin [20]); and other di-acids (e.g., succinic [9,18,21], maleic [1,16] or fumaric [22]); and this helps introduce extra functionality. Another method of functionalization is via a cross-linking agent and again a wide variety have been used, from straightforward dimethyl itaconate (DMI) [18] to highly functionalised *N*-alkylated dinitrones [21]. The methodology for the synthesis and subsequent cross-linking of the oligomers is relatively consistent throughout the literature; condensation reactions followed by radical polymerisation; with the exception of occasional use of enzyme catalysis [23,24] or acyclic diene metathesis [17]. The number-average molecular weight (*M*_N_) of the oligomers used in the polymer formation is again, relatively consistent and typically low; <1000 Da [9,10,11,18]. The synthesis of larger oligomers by poly-condensation has been hampered by the tendency of the itaconate double bond to itself act as a cross-linker and often, radical inhibitors are needed to enable efficient formation of even the smallest oligomers [1,13,17,20,24].

Very recently, the syntheses of much larger polyesters by poly-condensation have been reported and *M*_N_ values of >800 kDa achieved. Chanda *et al.* [14] carried out the transesterification of dibutyl itaconate (DBI) with several aliphatic diols and obtained polymers of 15–816 kDa. Winkler *et al.* [15] subsequently published a similar transesterification, this time of DMI, with aliphatic diols which produced smaller polymers with substantially lower polydispersities (*Pd_i_*) of <1.85. Both groups employ radical inhibitors to prevent the cross-linking of the itaconate double bond which they then functionalise via Michael addition reactions with thiols [14,15] or amines [14].

The work described here continues the emerging trend of transesterification of dialkyl itaconates with aliphatic diols to produce large UPEs. An important focus of this paper is on the detailed spectroscopic analysis of the isolated polymers and its use in the understanding of a key undesirable side reaction which occurs, hydroxyl addition across the double bond known as the Ordelt reaction (or saturation) [25,26,27,28,29,30]. The investigation into not only use of alternative di-acid monomers or alternative diols and polyols but also co-polymer synthesis is reported including the successful achievement of <96% itaconate incorporation into succinate co-polymers which is a significant improvement on the previously reported 76% [22].

## 2. Results and Discussion

**Scheme 1:**
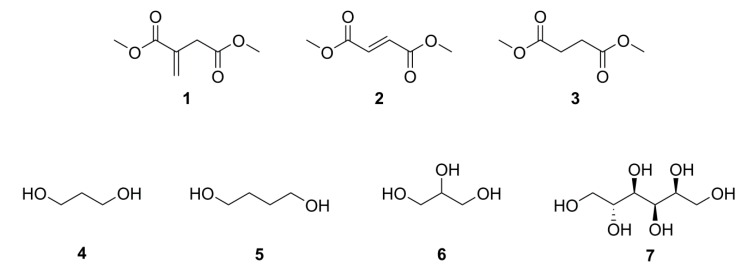
Bio-derived platform molecules **1**–**3** and various polyols **4**–**7** used for synthesis of unsaturated polyesters (UPEs). Dimethyl itaconate (DMI) **1**, dimethyl fumarate (DMFu) **2**, dimethyl succinate (DMS) **3**, 1,3-propanediol **4**, 1,4-butanediol **5**, glycerol **6** and sorbitol **7**. Conditions; Ti(IV)O*^t^*Bu_4_ catalyst, 175–200 °C, 29.5–68 h.

The synthesis of a range of UPEs from bio-derived platform molecules, Scheme 1, was successfully carried out and full and detailed analysis undertaken on the products. All diesters and diols and polyols used are obtainable from biomass via thermal, chemical or biological treatment of biomass within a bio-refinery facility [5].

### 2.1. Simple UPEs

**Scheme 2:**
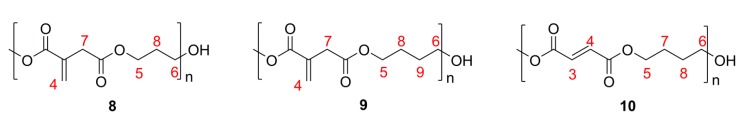
Annotated structures for poly(propylene itaconate) (PPI) **8**, poly(butylene itaconate) (PBI) **9** and poly(butylene fumarate) (PBFu) **10**. HA and HB are methyl groups of unsaturated and saturated itaconate esters respectively.

**Figure 1:**
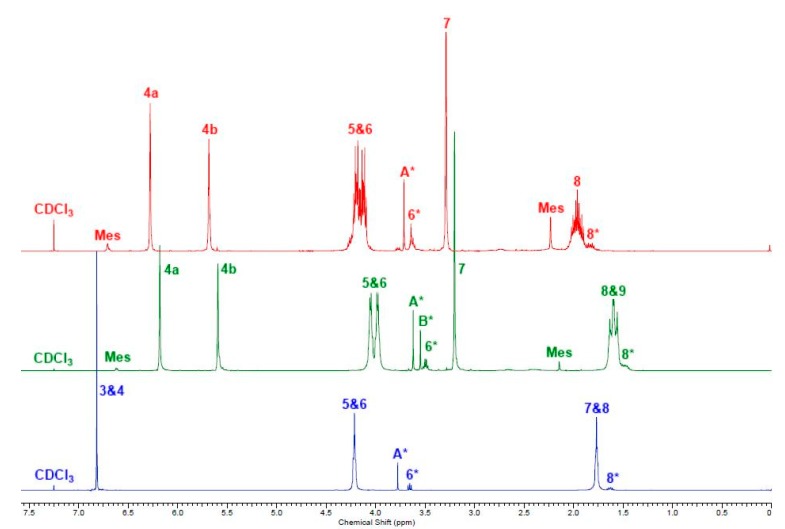
^1^H NMR spectra of PPI (red); PBI (green); PBFu (blue). The numbered signals (3 to 9) correspond to the protons assigned to the structures as shown in Scheme 2. A* and B* are the protons of the CH_3_ of the ester end-groups; 6* and 8* are the protons of the CH_2_ α and β to the hydroxyl end-groups respectively; 4a and 4b are for each one of the germinal protons on the itaconate double bond and are non-equivalent to one another; one is E to the ester group and the other is Z.

As can be seen by the ^1^H NMR spectra, Figure 1, the synthesis of the three UPEs; poly(propylene itaconate) (PPI), poly(butylene itaconate) (PBI) and poly(butylene fumarate) (PBFu), **8**, **9** and **10**, Scheme 2 respectively; was successful with the signals at 5.6 and 6.3 ppm (itaconate) and 6.8 ppm (fumarate) indicating the survival of the double bonds. ^1^H NMR spectroscopy end-group analysis suggested molecular weights of 2100, 1900 and 1800 Da for PPI, PBI and PBFu respectively; however it must be noted that signal resolution was poor as a result of broadening (indicative of polymeric samples).

Triple detection GPC analysis, Table 1, agreed well with the *M*_N_ predicted by ^1^H NMR spectra, confirming all three samples to be polymeric. A very high *M*_W_ material (*M*_N_ > 1 × 10^6^ Da), but of very low concentration (<0.5%), was observed in the itaconate samples and was removed from the following calculations but will be discussed later.

**Table 1:** GPC data for PPI, PBI and PBFu.

GPC Analysis	PPI	PBI	PBFu
*M*_N_	3600	580	2400
*M*_W_	10,600	2600	4300
*Pd*_i_		2.9	3.4

On closer inspection of the PPI and PBI ^1^H NMR spectra, signals at 2.2 and 6.7 ppm and similar signals previously observed in the literature have been attributed to the mesaconate moiety [22,31]. It was estimated by ^1^H NMR spectroscopy that 8% of the itaconate monomers had isomerised to the mesaconate equivalent. For fumarate containing UPEs no isomerisation to the maleate was observed.

**Scheme 3:**
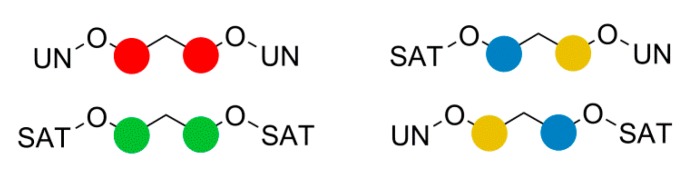
Saturated (SAT) and unsaturated (UNSAT) ester configurations for peaks H5 and H6 in PPI.

**Figure 2:**
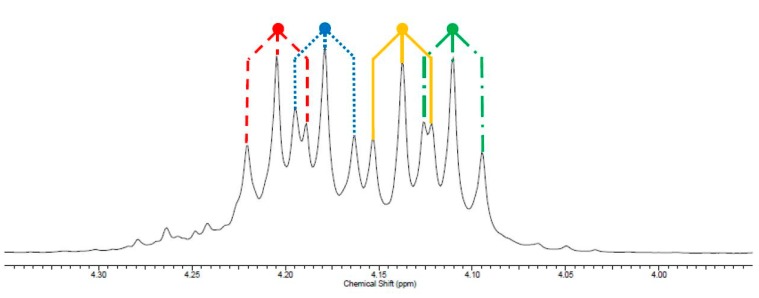
^1^H NMR spectra expansion of multiplicity for the peaks of the H5 and H6 region, showing assignment of four triplets as CH_2_s with UN–UN (red), SAT–SAT (green), UN–SAT (blue–yellow) and SAT–UN (yellow–blue) ester configuration. Assignments are speculatory.

An interesting, and unprecedented, observation from the ^1^H NMR spectra of PPI was the multiplicity of the signal assigned as H5 and H6 which was observed to be a doublet of doublet of triplets. 2D NMR spectroscopy showed no coupling of H5 to H6 or *vice versa* implying that the observed multiplicity was four separate triplets of equal intensity. It was rationalised that if saturated (SAT) and unsaturated (UN) esters of the itaconate moiety are considered as causing four possible environments for **5** and **6**, Scheme 3, each have an equal probability of occurring, Figure 2.

The same effect was also responsible for the apparent triplet of triplets seen for H8. The SAT-SAT and UN–UN arrangement for H8 would have given two quintets; however this was masked by the twice-as-probable UN–SAT and SAT–UN arrangement, giving the observed triplet of triplets (as H5 ≠ H6). The expected quintets for the UN–UN and SAT–SAT were most probably the cause of the broad nature of the region for H8.

Closer inspection of the ^13^C NMR spectra also supported this rationale as the region associated with the C8 had three peaks, with a rough 1:2:1 peak ratio. Analogous to the situation in the ^1^H NMR spectra the UN-UN and SAT-SAT were assigned as the two peaks with a relative integration of one, while the UN-SAT and SAT-UN gave the center peak with a relative integration of two. The two equal peaks for the C8* region, and four equal peaks for the C5 and C6 region, were also due to ester non-equivalence.

This same fine structure was also observed for PBI in both ^1^H and ^13^C NMR spectra but was less than seen for the PPI. This reduced effect was attributed to the additional CH_2_ of the diol chain reducing the interference of the other ester bond three atoms down.

High *M*_W_ and *Pd_i_* values for PPI and PBI were likely a result of chain branching and cross-linking, causing formation of exceptionally large polymers. Significant cross-linking was observed when performing polymerisation at elevated temperatures (180–220 °C); a viscosity limit for stirring was reached (2–10 h depending on temperature) and an opaque material was produced.

**Figure 3:**
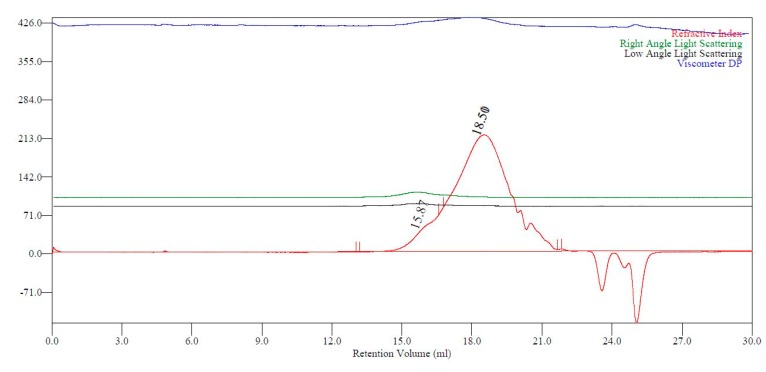
Triple detection GPC chromatogram for PBI backbone.

These opaque samples were insoluble in all solvents tested and it was therefore considered likely that the high molecular weight, low concentration, species observed by GPC, Figure 3, were a result of initial chain branching.

**Scheme 4:**
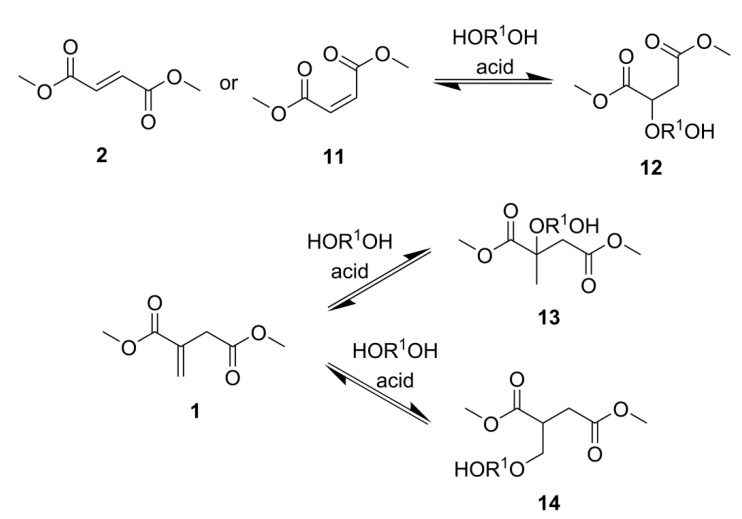
The Ordelt reaction, C=C bond saturation by a diol, of; DMFu or dimethyl maleate (DMM) **11** to compound **12** and DMI to compounds **13** and **14** via α- and β-addition respectively.

A conclusion of chain branching and eventual cross-linking has been postulated by other groups investigating UPE formation, especially when using DMI or IA as a substrate [31]. It is believed that the lateral C=C of itaconate has an increased activity compared to fumarate and maleate equivalents and, as such, can undergo spontaneous cross-linking. Literature suggests the mode of cross-linking was via the Ordelt reaction, an acid-catalysed C=C bond saturation by an alcohol [10,11,12,13,14,15]. According to the mechanism of the Ordelt reaction, proposed by Salmi *et al.* [28], hydroxyl adds at the α-position of the unsaturated ester. However, the model compounds synthesised to study C=C bond saturation were from fumarates and maleates, from which both *α-* and *β*-addition would have given the same product, **12**, Scheme 4 [27,28].

**Scheme 5:**
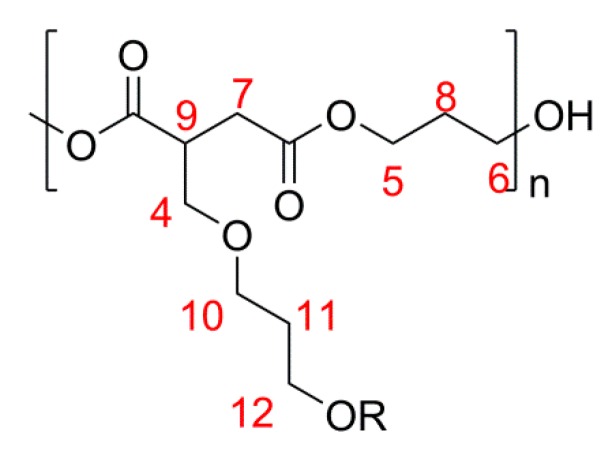
Annotated structure of branched saturated PPI.

**Figure 4:**
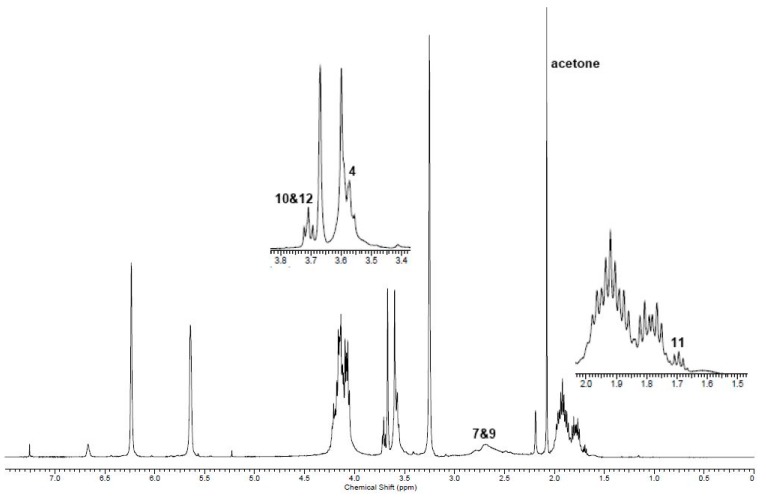
^1^H NMR spectrum for the branched unsaturated form of PPI. The numbered signals (4, 7 and 9) correspond to the protons assigned to the structures as shown in Scheme 5.

^1^H NMR spectroscopy analysis of the cross-linked material, Scheme 5 and Figure 4, formed during the attempted synthesis of PPI showed no CH_3_ (expected at 1.3–1.6 ppm) as a result of *α*-addition, **13**, Scheme 4. The appearance of the broad region from 2.5–3.0 ppm (H7 and H9), along with the increased complexity of the region at 3.7 ppm (H4, apparent triplet on shoulder of neighbouring peak), suggested that β-addition solely had occurred, **14**, Scheme 4; β-addition forms diastereotopic protons [32]. Peaks at 3.82 (H10 and H12, triplet, ^3^*J* = 5.68 Hz) and 1.80 (H11, quintet, ^3^*J* = 5.68 Hz) were attributed to the CH_2_s on the former diol section following ether formation. It should be noted that peaks for H10, H12 and H11 could also be present as a result of RO(CH_2_)_4_O(CH_2_)_4_OR ether linkages; however this would not explain the observation of branching and cross-linking. Signals from C=C saturation were too weak from the ^13^C NMR spectrum to be accurately assigned, although no obvious additional CH_3_s (from α-addition, expected at 20–30 ppm) were detected other than for isomerisation to mesaconate. By taking ratios of the ^1^H NMR signal for H11 and for the whole region of CH_2_s slightly downfield from this signal (*i.e.*, H8 and H8*) it was calculated that less than 4% of the diol added to the reaction was involved in C=C saturation.

The mechanism proposed by Salmi *et al.* [28] was for poly-esterification of a diacid, and not transesterification of a diester and therefore it may be possible that the mechanisms of saturation are different for esterification and transesterification reactions.

It was shown by Kharas *et al.* [32] that use of catalysts with greater acidity increases the extent of C=C saturation, and eventual cross-linking, in similar UPEs. Attempted polymerisation of IA with a diol formed an insoluble material and from the observations of Kharas *et al.* [32], it was considered highly probable that significant C=C saturation resulted in the cross-linked product catalysed by the inherent acidity of the IA monomer. This would imply that syntheses of UPEs containing itaconate within the backbone are most effectively achieved via transesterification, as demonstrated here.

NMR and IR spectroscopy analysis of the fumarate UPE, PBFu, indicated C=C saturation was less extensive compared to itaconate. Indeed, low *Pd_i_* for PBFu suggested that branching from C=C saturation was minimal, this being expected from the non-geminal C=C of fumarates. No low concentration/high mass species were observed in the PBFu sample, supporting the proposal that C=C saturation was the cause of this bi-modal anomaly in the GPC chromatogram for itaconate UPEs, whereby a small proportion of branched material was produced.

### 2.2. Co-UPEs

Incorporation of succinate into the backbone was studied with the aim of increasing backbone hydrophobicity however this would come at the expense of possible post-polymerisation modifications as the number of Michael accepting groups would decrease.

**Scheme 6:**
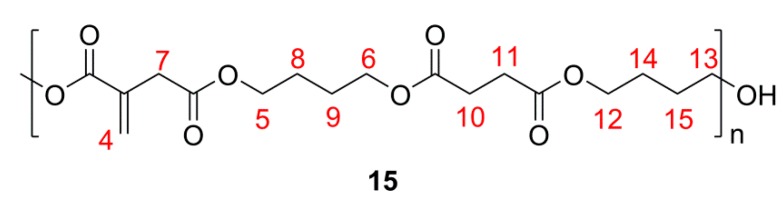
Annotated structure of poly(butylene itaconate-*co*-butylene succinate) (PBIBS) **15**. HA and HB are methyl groups of unsaturated and saturated itaconate esters respectively.

**Figure 5:**
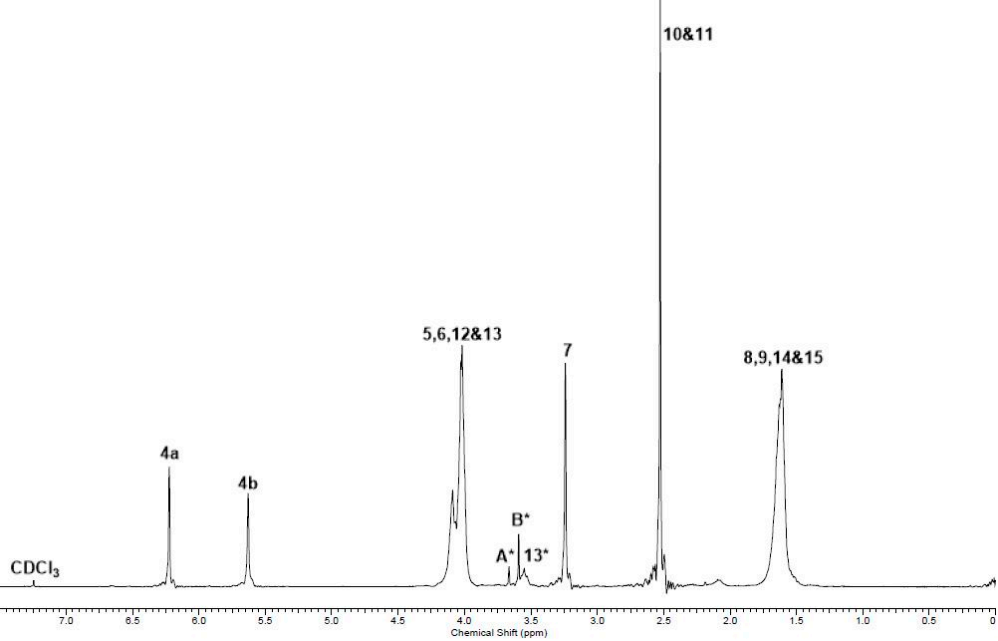
^1^H NMR of PBIBS. The numbered signals (4 to 15) correspond to the protons assigned to the structures as shown in Scheme 6. A* and B* are the protons of the CH_3_ of the ester end-groups; 13* are the protons of the CH_2_ α to the hydroxyl end-groups.

^1^H NMR analysis of poly(butylene itaconate-*co*-butylene succinate) (PBIBS), **15**, Scheme 6, confirmed the successful incorporation of dimethyl succinate (DMS) into the backbone and preservation of the C=C functionality, Figure 5. No evidence of isomerisation of the itaconate unit to the mesaconate or branching via the Ordelt reaction was observed from analysis of PBIBS. This was in agreement with observations of Sakuma *et al.* [33], suggesting that incorporation of succinate into the backbone is an effective way of reducing undesired isomerisation and chain branching.

**Scheme 7:**
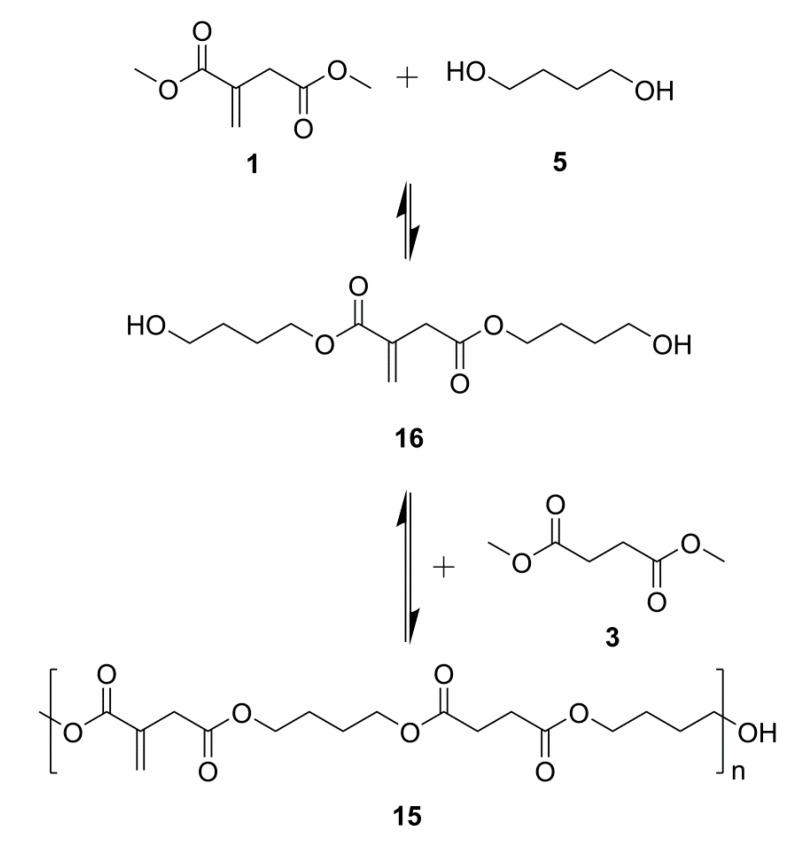
PBIBS synthesis via an intermediary *bis*-(4-hydroxyl butyl) itaconate **16**.

It was determined that 96% of the itaconate feed was eventually incorporated into the backbone by comparing the integration of H11 (itaconate) and H12 and H13 (succinate), Figure 5. This was a significant improvement on the %mol of itaconate incorporated by the methodology of either Sakuma *et al.* [33] (59%) or Teramoto *et al.* [22] (76%), where all reagents were added together at the start of the reaction. Evidently, our choice of prior formation of an intermediary *bis*-(4-hydroxyl butyl) itaconate, **16**, Scheme 7, was required in achieving the desired incorporation of itaconate and likely resulted in a significantly more ordered backbone of the *alt* notation.

PBIBS molecular weight distribution obtained from GPC, Table 2, was broader than seen for any of the simple UPEs, with a *Pd_i_* of 5.0 even after consideration of branching. The *M*_W_ would suggest that the polymer formed was of a larger mass than seen for the simple UPEs. The chromatogram was mono-modal, other than for the branching region, implying one type of polymer had formed (*i.e.*, no separate poly(butylene succinate)).

**Table 2:** GPC data for PBIBS.

GPC Analysis	Total Polymer (a)	High Conc./Low Mass (b)	Low Conc./High Mass (c)
*M*_N_	780	900	130,000
*M*_W_	69,000	8300	350,000
*Pd*_i_	85	5.0	2.8

### 2.3. Glycerol or Sorbitol UPEs

**Scheme 8:**
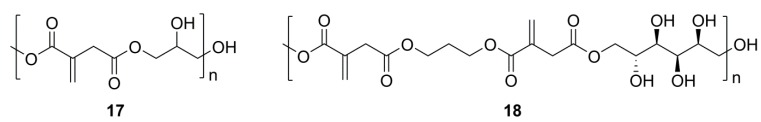
Structures of poly(glycerol itaconate) (PGI) **17** and poly(propylene itaconate-*co*-sorbitol itaconate) (PPISI) **18**.

Increasing the hydrophilicity of the UPE backbones was attempted by incorporation of glycerol and sorbitol. For poly(glycerol itaconate) (PGI), **17**, Scheme 8, glycerol was used as an exact molar replacement for 1,3-propanediol but for incorporation of sorbitol, poly(propylene itaconate-co-sorbitol itaconate) (PPISI), **18**, Scheme 8, only 50% of the 1,3-propanediol could be replaced as a higher feed of sorbitol resulted in a biphasic initial reaction mixture, even at elevated temperatures. It was hoped transesterification would be selective, limited only to primary alcohols however, complexity of the ^1^H NMR spectra of both PGI and PPISI suggested otherwise. Attempts to assign the spectra by advanced NMR techniques are shown in the experimental and the ^1^H NMR spectra are shown in the supplementary information, Figures S1 and S2.

Analysis of PGI and PPISI showed detectable levels of residual glycerol and 1,3-propanediol respectively, however quantification of these residues was impossible due to the complexity of the NMR spectra and broadness of the peaks. The low transesterification observed for glycerol and sorbitol monomers was likely due to the Ti(IV) catalyst center being excessively coordinated by the hydroxyls of these polyols, thereby preventing the ester of the itaconate also coordinating on the same Ti(IV). An excess of alcohol would prevent the formation of a catalytic species containing both alcohol and ester moieties, similar to the presence of acetylacetone, and thus reduce rates of transesterification.

**Scheme 9:**
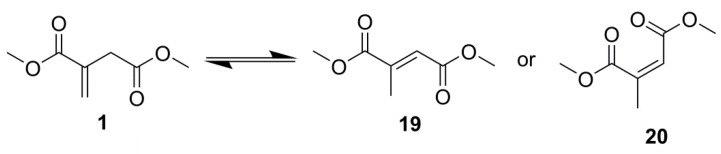
DMI isomerisation to dimethyl mesaconate **19** and dimethyl citraconate **20**.

^1^H NMR spectroscopy evidence showed extensive isomerisation, Table 3, to mesaconate and citraconate, **19** and **20** respectively, Scheme 9, higher than observed for previous polymerisations, however it was unclear whether this resulted from the presence of the extra hydroxyls or from the presence of magnesium oxide (MgO), necessary to increase transesterification yields.

**Table 3:** %mol itaconate:mesaconate:citraconate for the PGI and PPISI backbones.

Polymer	% Itaconate	% Mesaconate	% Citraconate
PGI	46	49	5
PPISI	57	39	4

Additionally the signals at 6.70 (mesaconate, 1H), 6.20–6.25 (itaconate, 1H), 6.04 (citraconate, 1H) and 5.81 (itaconate, 1H) were compared to the residual CH_3_ end group (3.71 ppm, 3H) to determine estimated levels of ester conversion, suggesting conversions of 89% and 79% for PGI and PPSIS respectively. Applying the Flory-Stockmayer theory points of gelation for PGI and PPSIS, under the ratios of reagents used, should have occurred at 86.6% and 41.0% conversion of the esters group respectively. It would therefore have been anticipated for both PGI and PPSIS samples prepared in this work to be insoluble gel, as conversions of the ester are above the critical gelation point. However, the Flory–Stockmayer theory assumes that each hydroxyl has equal reactivity, which in the case of glycerol and sorbitol is not true as the primary and secondary alcohols would show differing relative reactivities, therefore some deviation from the theoretical value is anticipated. The extensive side reactions (Ordelt saturation and isomerisation) could also have contributed to a deviation from the ideal scenario required for the Flory–Stockmayer model to apply. The PPSIS sample showed a strong deviation from the theoretical point of gelation, this could possibly have further been a result of the 50:50 mix of 1,3-propanediol to sorbitol and the 1,3-diol reacting preferentially, allowing higher esters conversion before significant branching from the sorbitol units has occurred.

GPC analysis of PGI (*M*_N_ = 2300, *M*_W_ = 6100, *Pd_i_* = 2.8) suggested that formation of polymeric material was successful. It is however possible that oligomers of glycerol could have been wrongly interpreted as larger linear polymers due to their hyper-branched structure as GPC is a volume separation, and not directly a measurement of mass. The oligomers, such as those described by Rollin *et al.* [34], may also be the reason for the significant complexity in the ^1^H NMR spectra, Figure S1, which supports the proposal of hyper-branching, and thus poor regioselectivity of the hydroxyls present (*i.e.*, there is no obvious selectivity between primary and secondary hydroxyls). Although several attempts were made, no suitable column/eluent system could be found for the PPISI material.

Both glycerol and sorbitol could be useful components in future biomass derivable UPEs for controlling polymer architecture, *i.e.*, for development of branching and cross-linking. However, from evidence shown it can be concluded that for uniform polymer architecture to be generated only small feeds of polyols should be used, especially as increased hydroxyl content adversely affects the activity of the Ti(IV) centered catalyst.

### 2.4. Advanced Analysis

Decomposition traces from thermo-gravimetric analysis (TGA) of the UPEs above were typically bi-modal however the initial decomposition was of significantly less mass loss and thus attributed to end-group decomposition. The possibility of residual monomer was discounted as DMI and vaporisation of polyols occurred at lower temperatures then those observed for the first mass losses in the polymer sample TGA traces.

Variations in temperature and ranges of decompositions of samples were observed. PPISI and PGI polymers possessed lower temperatures of decomposition with broader ranges which further exemplifies the diverse structure of these materials, Table 4.

**Table 4:** TGA temperature of decomposition (°C) and range of decomposition (°C) for the UPE backbones.

Backbone	1st Decomposition (Minor)	2nd Decomposition (Major)	Range of Decomposition
PPI	345.1	407.2	291–437
PBI		395.6	351–426
PBFu	258.7	381.8	337–425
PBIBS	304.5	399.3	333–413
PGI	280.3	336.8	273–446
PPISI		304.1	265–407

**Figure 6:**
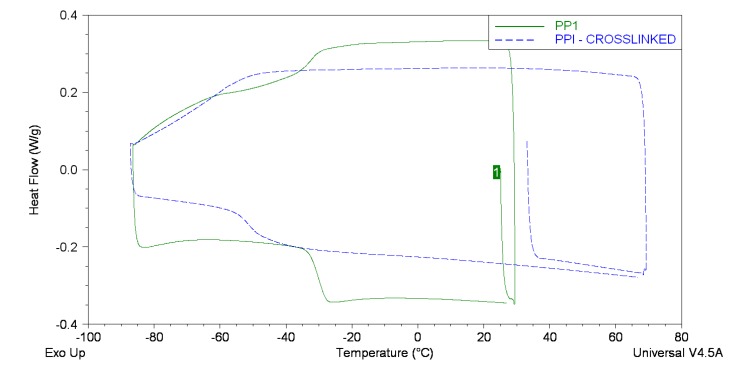
DSC Traces for PPI (solid line) and crosslinked PPI (dashed line).

Differential scanning calorimetry (DSC) was used to determine the glass transition temperatures (*T_g_*) of the UPEs, Table 5. PBIs lower *T*_g_ in comparison to PPI was probably due to increased flexibility as a result of the additional CH_2_ in the diol section. The *T*_g_ of PBIBS shows no deviation from PBI indicating that little extra chain flexibility was gained from succinate introduction. The cross-linked sample of PPI (~3% C=C saturation) had a *T*_g_ 21 °C lower than the linear equivalent. As shown by Figure 6 there is a distinct shift in the *T*_g_ on cross-linking as opposed to the appearance of two distinct glass transitions, therefore it is not that the soluble and insoluble portions of the sample are responsible for separate glass transitions. Normal convention would expect the *T*_g_ to rise on cross-linking as chain mobility is reduced. However it may be possible that, for the “cross-linked PPI” sample, the majority of the C=C saturation caused branching and not chain cross-linking. As branching forces chains further apart and disrupts the molecular packing the effect of plasticisation (*i.e.*, lowering of *T*_g_) can be observed [22]. PGI and PPISI possessed the highest *T*_g_s which was likely caused by extensive chain branching or chain-chain hydrogen bonding reducing the ease of chain rotation and flexing.

No *T*_g_ was detected for PBFu over the range studied (−80 to 200 °C), although a definite melting temperature (m.p. = 135.4 °C) was seen. The lack of a *T*_g_ was indicative of a highly crystalline material possessing a low amorphous content [35]. Kharas *et al.* [32] indicated the presence of glass transitions for fumarate UPEs (−6 to 23 °C) although all the diols used in Kharas’ study were non-linear (e.g., 1,2-propanediol) and thus more likely to yield amorphous regions.

The other backbones did not exhibit a melting point, with the exception of PBIBS, where slight deformations of the baseline at 20 to 60 °C could indicate some crystallisation and subsequent melting.

**Table 5:** *T*_g_ of UPE backbones. @, *T*_g_ not observed, value is *T*_m_.

Backbone	*T*_g_/°C	Range/°C
PPI	−30.1	−32.8 to −28.0
PPI (crosslinked)	−51.2	−54.3 to −48.9
PBI	−41.7	−44.8 to −39.6
PBFu ^@^	135.4	112 to 138
PBIBS	−41.3	−43.5 to −39.8
PGI	−20.9	−25.5 to −16.4
PPISI	−16.6	−21.0 to −11.8

### 2.5. Solubilities

**Figure 7:**
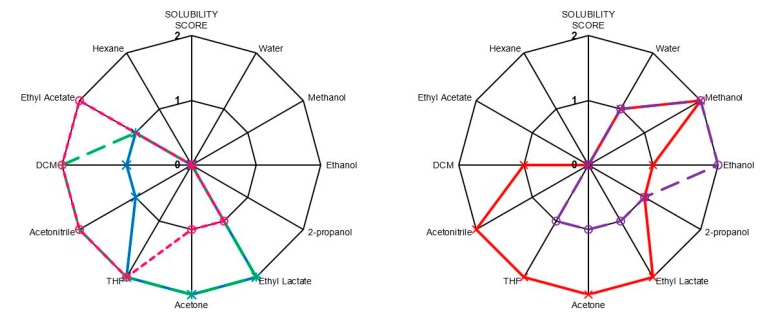
Solubility plots for UPEs; blue = PPI, green = PBI, pink = PBIBS, red = PGI and purple = PPISI; 0 = no solubility, 1 = moderate solubility and 2 = high solubility.

Figure 7 demonstrates variation of solubility of the different polymer backbones in a range of solvents. PGI and PPISI were most soluble in hydrophilic solvents such as water and methanol, while PBIBS was more soluble in hydrophobic solvents such as DCM and ethyl acetate. PPI and PBI demonstrated very similar solubility, the only difference being increased solubility in DCM and ethyl acetate when using 1,4-butanediol monomer.

PBFu was found to be insoluble in nearly all solvents investigated. This may be attributed to its high crystallinity (as shown by DSC—no *T*_g_ observed). It could be expected that the incorporation of a small amount of itaconate in place of fumarate would improve the solubility of the PBFu backbone by introducing irregularity which would allow solvent to penetrate the structure and force the chains apart.

## 3. Experimental Section

### 3.1. Materials and Instrumentation

DMI and DMS were purchased from Fluka (Buchs, Switzerland); dimethyl fumarate (DMFu), 1,3-propanediol, Ti(IV) tetra-*tert*-butoxide, dimethyl maleate (DMM), ethyl lactate, magnesium oxide, DMSO-*d*_6_ and CDCl_3_ were purchased from Sigma-Aldrich (St. Louis, MO, USA); glycerol and all solvents were purchased from Fisher Scientific (Hampton, NH, USA).

^1^H and proton-decoupled ^13^C NMR spectra were obtained on a Jeol 400 spectrometer (Akishima, Tokyo, Japan), in various deuterated solvents. Chemical shifts were calibrated using the internal solvent resonance and referenced to TMS. IR spectra were obtained on a Bruker Vertex 70 (Billerica, MA, USA) fitted with Specac Golden Gate ATR. Organic polymer solutions (10 mg·mL^−1^ polymer in eluent) were analysed by GPC using a Malvern Instruments Viscotek system employing OmniSEC 4.2 software (Malvern, Worcestershire, UK), TDA Model 302 (right-angled and low-angled light scattering, refractive index, ultra-violet and viscometer detectors all in a temperature controlled oven (35 °C)) coupled to a gpcMAX integrated solvent and sample delivery module (degasser, pump and autosampler). The system was fitted with two GMPWx1 + guard columns (35 °C), at a flow rate of 1 mL·min^−1^, using either THF or CHCl_3_ as the eluent, and with an injection volume of 100 μL. All samples analysed by GPC were ensured to be totally soluble in THF prior to filtration, any with incomplete solubility were disregarded for GPC analysis as these would not give representative results. For the TA Instruments Q-series TGA system (New Castle, DE, USA), ~10 mg of sample was used and 25 mL·min^−1^ of N_2_ gas was passed through the furnace for the duration of the analysis, with a balance gas flow of 10 mL·min^−1^. All samples were heated from room temperature to 700 °C at a rate of 10 °C·min^−1^. Differential scanning calorimetry (DSC) was performed on a Q-series TA Instruments DSC (New Castle, DE, USA). A heat-cool-heat program was used, with ~4 mg sample. Values for glass transition temperature (*T*_g_) were taken as the point of inflection (*I*), with the temperature range as the start and end of the slope on which I lies. When quoted, the melting temperature (*T*_m_) and crystallisation temperature (*T*_c_) were taken at the point of lowest and highest heat flow respectively. The melting and crystallisation ranges were the points where deviation from the baseline began and ended. The onset temperature of *T_m_* and *T*_c_ were calculated from extrapolation of the phase change slope to the baseline.

### 3.2. Synthesis of UPEs

#### 3.2.1. Simple UPEs

DMI or dimethyl fumarate (DMFu) and 1,3-propanediol or 1,4-butanediol (1 mol equiv.) were stirred together (magnetic stirrer flea, 500 rpm) and gradually heated in a three-neck round bottomed flask, with a Dean-Stark condenser attached, in an oil bath set to 175 °C. Once the oil had reached a temperature of 165 °C Ti(IV) tetra-*tert*-butoxide (1.5 wt %) was added via a syringe. After 9 h the temperature set-point was increased to 200 °C. After a further 24 h the reaction was subsequently put under a ~5 mbar vacuum for a further 15 h. After cooling, a highly viscous yellow transparent resin was collected.

PPI ^1^H NMR (400 MHz, CDCl_3_, δ_H_, ppm): 1.81 (0.2H, m, [RCO_2_CH_2_C*H_2_*CH_2_]_n_OH, end-group), 1.94 (2H, 2t, ^3^*J* = 6.1 Hz, [RCO_2_CH_2_C*H_2_*CH_2_]_n_OH), 2.22 (0.24H, s, CH_3_[CO_2_C(C*H_3_*)=CHCO_2_R]_n_, mesaconate), 3.27 (2H, s, CH_3_[CO_2_C(=CH_2_)C*H_2_*CO_2_R]_n_OH), 3.62 (0.2H, br m, [RCO_2_CH_2_CH_2_C*H_2_*]_n_OH, end-group), 3.70 (0.2H, s, C*H_3_*[CO_2_C(=CH_2_)CH_2_CO_2_R]_n_OH, end-group), 4.15 (4H, 4 t, ^3^*J =* 6.1 Hz, CH_3_[RCO_2_C*H_2_*CH_2_C*H_2_*]_n_OH), 5.67 (1H, s, CH_3_[CO_2_C(=C*H*H)CH_2_CO_2_R]_n_OH), 6.26 (1H, s, CH_3_[CO_2_–C(=CH*H*)CH_2_CO_2_R]_n_OH) 6.69 (0.08H s, CH_3_[CO_2_C(CH_3_)=C*H*CO_2_R]_n_, mesaconate); ^13^C NMR (100 MHz, CDCl_3_, δ_C_, ppm): 14.2 (~0.05C, CH_3_[CO_2_C(*C*H_3_)=CHCO_2_R]_n_, mesaconate), 27.7 (3 peaks 1:2:1, [RCO_2_–CH_2_*C*H_2_CH_2_]_n_OH), 31.5 (2 equal peaks, [RCO_2_CH_2_*C*H_2_CH_2_]_n_OH, end-group), 37.5 ([RCO_2_C(=CH_2_)*C*H_2_–CO_2_R]_n_), 52.1 (~0.07C, RCO_2_*C*H_3_, end-group), 58.8 (~0.11C, 2 equal peaks, [RCO_2_CH_2_–CH_2_*C*H_2_]_n_OH), 61.4 (2C, 4 equal peaks, [RCO_2_*C*H_2_CH_2_*C*H_2_]_n_OH), 61.9 (~0.1C, [RCO_2_*C*H_2_CH_2_CH_2_]_n_OH, end-group), 126.4 (~0.05C, CH_3_[CO_2_C(CH_3_)=*C*HCO_2_R]_n_, mesaconate), 128.7 ([RCO_2_C(=*C*H_2_)CH_2_–CO_2_R]_n_), 133.4 ([RCO_2_*C*(=CH_2_)CH_2_CO_2_R]_n_), 143.7 (~0.03C, CH_3_[CO_2_*C*(CH_3_)=CHCO_2_R]_n_, mesaconate), 165.5 (R*C*O_2_R, mesaconate), 165.8 ([R*C*O_2_C(=CH_2_)CH_2_CO_2_R]_n_), 165.9 (shoulder of previous peak, (CH_3_[*C*O_2_C(=CH_2_)CH_2_–CO_2_R]_n_, end-group), 166.5 (R*C*O_2_R, mesaconate), 170.4 ([RCO_2_C(=CH_2_)CH_2_–*C*O_2_R]_n_), 170.5 (shoulder of previous peak, ([RCO_2_C(=CH_2_)CH2*C*O_2_]_n_CH_3_, end-group); IR (*ν*, cm^−1^), 2966 (C–H), 2903 (C–H), 1713 (C=O, ester), 1639 (C=C).

PBI ^1^H NMR (400 MHz, CDCl_3_, δ_H_, ppm): 1.47 (0.22H, m, [RCO_2_CH_2_CH_2_C*H_2_*CH_2_]_n_OH, end-group), 1.59 (4H, br m, [RCO_2_CH_2_C*H_2_*C*H_2_*CH_2_]_n_OH), 2.13 (~0.06H, s, CH_3_[CO_2_C(C*H_3_*)=CHCO_2_R]_n_, mesaconate), 3.19 (2H, s, CH_3_[CO_2_C(=CH_2_)C*H_2_*CO_2_R]_n_OH), 3.48 (0.24H, apparent q, likely 2t, [RCO_2_CH_2_CH_2_–CH_2_C*H_2_*]_n_OH), end-group), 3.61 (0.24H, s, C*H_3_*[CO_2_C(=CH_2_)CH_2_CO_2_R]_n_OH), end-group), 4.01 (4H, br m, [RCO_2_C*H_2_*CH_2_CH_2_C*H_2_*]_n_OH), 5.58 (1H, s, CH_3_[CO_2_C(=C*H*H)CH_2_CO_2_R]_n_OH), 6.17 (1H, s, CH_3_[CO_2_–C(=CH*H*)CH_2_CO_2_R]_n_OH), 6.61 (~0.02H, s, CH_3_[CO_2_C(CH_3_)=C*H*CO_2_R]_n_, mesaconate); ^13^C NMR (100 MHz, CDCl_3_, δ_C_, ppm): 14.2 (~0.05C, CH_3_[CO_2_C(*C*H_3_)=CHCO_2_R]_n_, mesaconate), 24.8 (2C, 3 peaks 1:2:1, [RCO_2_CH_2_*C*H_2_*C*H_2_CH_2_]_n_OH), 28.6 (~0.14C, 2 equal peaks, [RCO_2_CH_2_–CH_2_*C*H_2_CH_2_]_n_OH, end-group), 37.1 (~0.14C, [RCO_2_CH_2_*C*H_2_CH_2_CH_2_]_n_OH end-group), 37.3 ([RCO_2_–C(=CH_2_)*C*H_2_CO_2_R]_n_), 51.6 (~0.15C, 2 equal peaks, RCO_2_*C*H_3_, end-group), 61.2 (~0.17C, [RCO_2_CH_2_–CH_2_CH_2_*C*H_2_]_n_OH, end-group), 63.9 (2C, 2 equal peaks, [RCO_2_*C*H_2_CH_2_CH_2_*C*H_2_]_n_OH), 64.2 (~0.14C, 2 equal peaks, [RCO_2_*C*H_2_CH_2_–CH_2_CH_2_]_n_OH, end-group), 126.1 (~0.05C, CH_3_[CO_2_C(CH_3_)=*C*HCO_2_R]_n_, mesaconate), 128.1 ([RCO_2_–C(=*C*H_2_)CH_2_CO_2_R]_n_), 133.5 ([RCO_2_*C*(=CH_2_)CH_2_CO_2_R]_n_), 143.7 (~0.02C, CH_3_[CO_2_*C*(CH_3_)=CH–CO_2_R]_n_, mesaconate), 165.3 (R*C*O_2_R, mesaconate), 165.7 ([R*C*O_2_C(=CH_2_)CH_2_–CO_2_R]_n_), 166.2 (CH_3_[*C*O_2_–C(=CH_2_)CH_2_CO_2_R]_n_, end-group), 166.5 (R*C*O_2_R, mesaconate), 170.3 ([RCO_2_C(=CH_2_)CH_2_*C*O_2_R]_n_), 170.7 ([RCO_2_C(=CH_2_)CH2*C*O_2_]_n_CH_3_, end-group); IR (*ν*, cm^−1^), 2959 (C–H), 2899 (C–H), 1712 (C=O, ester), 1638 (C=C).

PBFu ^1^H NMR (400 MHz, CDCl_3_, δ_H_, ppm): 1.67 (0.24H, m, [RCO_2_CH_2_CH_2_C*H_2_*CH_2_]_n_OH, end-group), 1.81 (4H, br m, [RCO_2_CH_2_C*H_2_*C*H_2_*CH_2_]_n_OH), 3.70 (0.21H, t, ^3^*J* = 6.4 Hz, [RCO_2_CH_2_CH_2_CH_2_–C*H_2_*]_n_OH), end-group), 3.82 (0.28H, s, C*H_3_*[CO_2_CH=CHCO_2_R]_n_OH), end-group), 4.23 (4H, br m, [RCO_2_C*H_2_*CH_2_–CH_2_C*H_2_*]_n_OH), 6.85 (0.25H, s, CH_3_[CO_2_C*H*=CHCO_2_R]_n_OH or CH_3_[CO_2_CH=C*H*–CO_2_R]_n_OH, end-group), 6.86 (2H, s, CH_3_[CO_2_C*H*=C*H*CO_2_R]_n_OH), 6.87 (0.23H, s, CH_3_[CO_2_C*H*=CH–CO_2_R]_n_OH or CH_3_[CO_2_–CH=C*H*CO_2_R]_n_OH, end-group); ^13^C NMR (100 MHz, CDCl_3_, δ_C_, ppm): 25.0 (~0.11C, [RCO_2_CH_2_*C*H_2_–CH_2_CH_2_]_n_OH, end-group), 25.2 (2C, [RCO_2_CH_2_*C*H_2_*C*H_2_CH_2_]_n_OH), 29.0 (~0.11C, [RCO_2_CH_2_CH_2_*C*H_2_–CH_2_]_n_OH end-group), 52.4 (~0.1C, RCO_2_*C*H_3_, end-group), 62.2 (~0.11C, [RCO_2_CH_2_CH_2_CH_2_*C*H_2_]_n_OH), 64.7 (2C, [RCO_2_*C*H_2_CH_2_CH_2_*C*H_2_]_n_OH), 65.2 (~0.11C, [RCO_2_*C*H_2_CH_2_CH_2_CH_2_]_n_OH end-group), 133.4 (~0.15C, CH_3_[CO_2_*C*H=CHCO_2_R]_n_ or CH_3_[CO_2_CH=*C*H–CO_2_R]_n_, end-group), 133.6 (2C, CH_3_[CO_2_–*C*H=C*H*CO_2_R]_n_), 133.8 (~0.12C, CH_3_[CO_2_*C*H=CHCO_2_R]_n_ or CH_3_[CO_2_CH=*C*HCO_2_R]_n_, end-group), 164.8 (2C, [R*C*O_2_CH=CH*C*O_2_R]_n_), 165.4 (~0.07C, likely CH_3_[*C*O_2_CH=CHCO_2_R]_n_, end-group); IR (*ν*, cm^−1^), 2952 (C–H), 2860 (C–H), 1703 (C=O, ester), 1663 (C=C), 1468, 1447.

#### 3.2.2. PBIBS

DMI and 1,4-butanediol (2 mol equiv.) were stirred together (magnetic stirrer flea, 500 rpm) and gradually heated in a three-neck round bottomed flask, with a Dean–Stark condenser attached, in an oil bath set to 175 °C. Once the oil had reached a temperature of 150 °C Ti(IV) tetra-*tert*-butoxide (0.6 wt %) was added via a syringe. After 1.5 h DMS (1 mol equiv.) was added drop-wise over the course of 3 h. After 22 h the reaction was gradually put under a ~5 mbar vacuum for a further 3 h. On cooling a highly viscous yellow transparent resin was collected.

^1^H NMR (270 MHz, CDCl_3_, δ_H_, ppm): 1.61 (8H, br, [RCO_2_CH_2_C*H_2_*C*H_2_*CH_2_]_n_OH), 2.53 (4H, br, CH_3_[CO_2_C*H_2_*C*H_2_*CO_2_R]_n_OH), 3.24 (1.86H, s, CH_3_[CO_2_C(=CH_2_)C*H_2_*CO_2_R]_n_OH), 3.56 (0.36H, br m, [RCO_2_CH_2_CH_2_CH_2_C*H_2_*]_n_OH), end-group), 3.65 (0.14H, s, C*H_3_*[CO_2_R]_n_OH, end-group), 4.03 (8H, br, [RCO_2_C*H_2_*CH_2_CH_2_C*H_2_*]_n_OH), 5.63 (0.93H, s, CH_3_[CO_2_C(=C*H*H)CH_2_CO_2_R]_n_OH), 6.23 (0.93H, s, CH_3_[CO_2_C(=CH*H*)CH_2_CO_2_R]_n_OH); ^13^C NMR (67.5 MHz, CDCl_3_, δ_C_, ppm): 24.5 (4C, [RCO_2_CH_2_–*C*H_2_*C*H_2_CH_2_]_n_OH), 28.8 (2C, CH_3_[CO_2_C*H_2_*C*H_2_*CO_2_R]_n_OH), 37.4 (1C, [RCO_2_C(=CH_2_)*C*H_2_CO_2_R]_n_), 50.1 (~0.13C, 3 peaks 1:1:2, RCO_2_*C*H_3_, end-group), 61.8 (~0.18C, [RCO_2_CH_2_CH_2_*C*H_2_]_n_OH), 64.1 (4C, 3 peaks 1:1:2, [RCO_2_*C*H_2_CH_2_CH_2_*C*H_2_]_n_OH), 128.2 ([RCO_2_C(=*C*H_2_)CH_2_CO_2_R]_n_), 133.6 ([RCO2–*C*(=CH_2_)CH_2_–CO_2_R]_n_), 165.8 (1C,[R*C*O_2_C(=CH_2_)CH_2_CO_2_R]_n_), 170.4 (1C, [RCO_2_C(=CH_2_)CH_2_*C*O_2_R]_n_), 172.0 (2C,[R*C*O_2_–CH_2_CH_2_*C*O_2_]_n_CH_3_); IR (*ν*, cm^−1^), 2961 (C–H), 1715 (C=O, ester), 1638 (C=C).

#### 3.2.3. PGI

DMI and glycerol (1 mol equiv.) were stirred together (magnetic stirrer flea, 1200 rpm) and gradually heated in a three-neck round bottomed flask, with a Dean-Stark condenser attached, in an oil bath set to 175 °C. Once the oil had reached a temperature of 165 °C Ti(IV) tetra-*tert*-butoxide (0.4 wt %) was added via a syringe. After 48 h MgO (0.1 wt %, freshly calcined, 800 °C) was added and the reaction was gradually put under a 50 mbar vacuum for 10 h and 30 mbar for a further 10 h. On cooling a toffee-like material was collected.

Due to the complexity of the spectra integration was impossible for the majority of the peaks assigned. HSQC, COSY and DEPT were required for assignment; ^1^H NMR (400 MHz, 60 °C, *d*_6_-DMSO, δ_H_, ppm): 2.00 (0.3H, s, CH_3_[CO_2_C(C*H_3_*)=CHCO_2_R]_n_, citraconate), 2.13 (3H, s, CH_3_[CO_2_C(C*H_3_*)=CH–CO_2_R]_n_, mesaconate), 2.4–2.9 (br m, CH_3_[CO_2_C*H*(CH_2_OCH_2_CH(OR)CH_2_OR)C*H_2_*CO_2_R]_n_OH, Ordelt), 3.3–3.45 (br m, RC*H_2_*OH and CH_3_[CO_2_C(=CH_2_)C*H_2_*CO_2_R]_n_OH), 3.45–3.8 (numerous peaks, br, CO_2_C*H_3_* and CH_2_C*H*(OH)CH_2_), 3.9–4.4 (numerous peaks, br, ROC*H_2_*CH(OR)C*H_2_*OR and ROCH_2_–CH(OH)C*H_2_*OR and ROC*H_2_*CH(OR)CH_2_OH), 4.4–5.3 (numerous peaks, br, disappears on D_2_O shake, CO*H*), 5.81 (1H, s, CH_3_[CO_2_C(=C*H*H)CH_2_CO_2_R]_n_OH), 6.04 (0.1H, s, CH_3_[CO_2_C(CH_3_)=C*H*CO_2_R]_n_, citraconate), 6.20 (0.55H, CH_3_[CO_2_C(=CH*H*)CH_2_CO_2_R]_n_OH, end-group or main chain), 6.25 (0.45H, CH_3_[CO_2_C(=CH*H*)CH_2_–CO_2_R]_n_OH, end-group or main chain), 6.70 (1H, br, CH_3_[CO_2_C(CH_3_)=C*H*–CO_2_R]_n_, mesaconate); ^13^C NMR (100 MHz, 60 °C, *d*_6_-DMSO, δ_C_, ppm): 13.9 (CH_3_[CO_2_C(*C*H_3_)=CH–CO_2_R]_n_, mesaconate), 19.5 (CH_3_[CO_2_C(*C*H_3_)=CHCO_2_R]_n_, citraconate), 39.2 ([RCO_2_C(=CH_2_)*C*H_2_–CO_2_R]_n_), 51.6 (3 peaks, RCO_2_*C*H_3_), 62.6 (R*C*H_2_OH), 63.1 (R*C*H_2_OH), 66.1 (several peaks, R*C*H_2_COC(=O)R), 69.1 (2 peaks, R*C*H(OH)R), 72.3 (R*C*H(OH)R, potentially glycerol), 112.9 (weak, CH_3_[CO_2_C(CH_3_)=*C*HCO_2_R]_n_, citraconate), 126.0 (CH_3_[CO_2_C(CH_3_)=*C*HCO_2_R]_n_, mesaconate), 128.3 ([RCO_2_C(=*C*H_2_)CH_2_CO_2_R]_n_), 133.6 ([RCO_2_*C*(=CH_2_)CH_2_CO_2_R]_n_), 142.7 (CH_3_[CO_2_*C*(CH_3_)=CHCO_2_R]_n_, mesaconate), 164–167 (numerous peaks, R*C*O_2_R), 169–171 (numerous peaks, R*C*O_2_R); IR (*ν*, cm^−1^), 3431 (broad, O–H), 2955 (C–H), 2900 (C–H), 1715 (C=O, ester), 1645 (C=C), 1438 (H–C–H, residual methyl ester).

#### 3.2.4. PPISI

DMI, 1,3-propanediol (0.5 mol equiv.) and D-sorbitol (0.5 mol equiv.) were stirred together (magnetic stirrer flea, 1200 rpm) and gradually heated in a three-neck round bottomed flask, with a Dean–Stark condenser attached, in an oil bath set to 175 °C. Once the oil had reached a temperature of 165 °C Ti(IV) tetra-*tert*-butoxide (0.6% wt) was added via a syringe. After 48 h MgO (0.25 wt %, freshly calcined, 800 °C) was added and the reaction was gradually put under a 5 mbar vacuum for a further 10 h. On cooling a toffee-like material was collected.

Due to the complexity of the spectra integration was impossible for the majority of the peaks assigned. HSQC, COSY and DEPT were required for assignment; ^1^H-NMR (400 MHz, 60 °C, *d*_6_-DMSO, δ_H_, ppm, integration relative to central CH_2_ of diol end-group): 1.58 (~0.3H, quin, ^3^*J* = 6.3 Hz, HOCH_2_C*H_2_*–CH_2_OH), 1.75 (1H, m, [RCO_2_CH_2_C*H_2_*CH_2_]_n_OH, end-group), 1.94 (~0.25H, m, [RCO_2_CH_2_C*H_2_*–CH_2_]_n_OH), 2.00 (~0.15H, s, CH_3_[CO_2_C(C*H_3_*)=CHCO_2_R]_n_, citraconate), 2.18 (~0.8H, s, CH_3_[CO_2_C(C*H_3_*)=CH–CO_2_R]_n_, mesaconate), 2.4–2.9 (br m, CH_3_[CO_2_C*H*(CH_2_OCH_2_R)C*H_2_*CO_2_R]_n_OH, Ordelt), 3.35 (m, shoulder of neighbouring peak, CH_3_[CO_2_C(=CH_2_)C*H_2_*CO_2_R]_n_OH), 3.4–3.8 (numerous peaks, br, CO_2_C*H_3_* and C*H*OH and HOC*H_2_*CH_2_C*H_2_*OH), 3.95 (br m, disappears on D_2_O shake, *H*OCH_2_CH_2_CH_2_O*H*), 3.9–4.2 (numerous peaks, br, RC*H_2_*OH and ROC*H_2_*CH(OH)R and R_2_C*H*OH), 4.25 (m, ROCH_2_CH_2_C*H_2_*OH, end-group), 4.36 (br m, ROC*H_2_*CH_2_C*H_2_*OR), 4.6–5.2 (numerous peaks, br, disappears on D_2_O shake, CO*H*), 5.80 (~0.23H, s, CH_3_[CO_2_C(=C*H*H)CH_2_CO_2_R]_n_OH), 6.02 (~0.02H, s, CH_3_[CO_2_C(CH_3_)=C*H*CO_2_R]_n_, citraconate), 6.19 (~0.23H, CH_3_[CO_2_C(=CH*H*)CH_2_CO_2_R]_n_OH,), 6.64 (~0.13H, br, CH_3_[CO_2_C(CH_3_)=C*H*–CO_2_R]_n_, mesaconate); ^13^C NMR (100 MHz, 60 °C, *d*_6_-DMSO, δ_C_, ppm): 13.8 (CH_3_[CO_2_C(*C*H_3_)=CH–CO_2_R]_n_, mesaconate), 27.4 (weak, ROCH_2_*C*H_2_CH_2_OR), 31.4 (ROCH_2_–*C*H_2_CH_2_OH), 35.6 (HOCH_2_*C*H_2_CH_2_OH), 37.0 (several peaks, [RCO_2_C(=CH_2_)*C*H_2_CO_2_R]_n_), 51.6 (several peaks, RCO_2_*C*H_3_), 57.2 (ROCH_2_CH_2_*C*H_2_OH), 58.1 (HO*C*H_2_CH_2_*C*H_2_OH), 60–62 (~6 peaks, *C*H_2_OH, sorbitol section), 69.5–80.5 (~20 peaks, *C*HOH and *C*HOR, sorbitol section), 125.9 (CH_3_[CO_2_–C(CH_3_)=*C*HCO_2_R]_n_, mesaconate), 128.2 ([RCO_2_C(=*C*H_2_)CH_2_CO_2_R]_n_), 133.7 ([RCO_2_*C*(=CH_2_)CH_2_–CO_2_R]_n_), 142.8 (CH_3_[CO_2_–*C*(CH_3_)=CHCO_2_R]_n_, mesaconate), 164–167 (numerous peaks, R*C*O_2_R), 169–171 (numerous peaks, R*C*O_2_R); IR (*ν*, cm^−1^), 3433 (broad, O–H), 2954 (C–H), 1722 (C=O, ester), 1645 (C=C), 1436 (H–C–H, residual methyl ester).

## 4. Conclusions

This paper reports the successful synthesis of UPEs with tunable solubilities via poly-transesterification of bio-derived platform molecules with various diols. Extensive analytical investigations revealed important, previously unreported, structural characteristics of the UPEs and highlighted two undesired side reactions of the polymerisations; the isomerisation of itaconate units to mesaconate and citraconate, and the saturation of C=Cs (Ordelt reaction).

Co-UPEs of itaconates and succinates were successfully formed with significantly higher itaconate incorporation than previously reported in the literature [22,33] and these polymers showed decreased side reactions and increased solubility in less polar solvents than the UPEs. Replacing diols for polyols yielded complex polymers with highly branched backbones and inter-chain hydrogen bonding capabilities, increasing *T_g_* and solubility in polar solvents.

The results presented here can not only be used to aid the synthesis of larger polyesters retaining the *exo*-unsaturated functionality but also to widen the variety of co-polymers obtainable via poly-transesterification both in terms of monomer range and percent composition of itaconates, ultimately effecting the polarity and solubility of the polymers in various solvents.

## Supplementary Materials

Supplementary materials can be found at http://www.mdpi.com/1422-0067/16/07/14912/s1.
