# Supplementary figures and images for: Synthesis of Unsaturated Polyester Resins from Various Bio-Derived Platform Molecules
Source: Int J Mol Sci. 2015 Jul 2;16(7):14912–32. doi: 10.3390/ijms160714912 (PMC4519879; doi:10.3390/ijms160714912)

## Supplementary Information

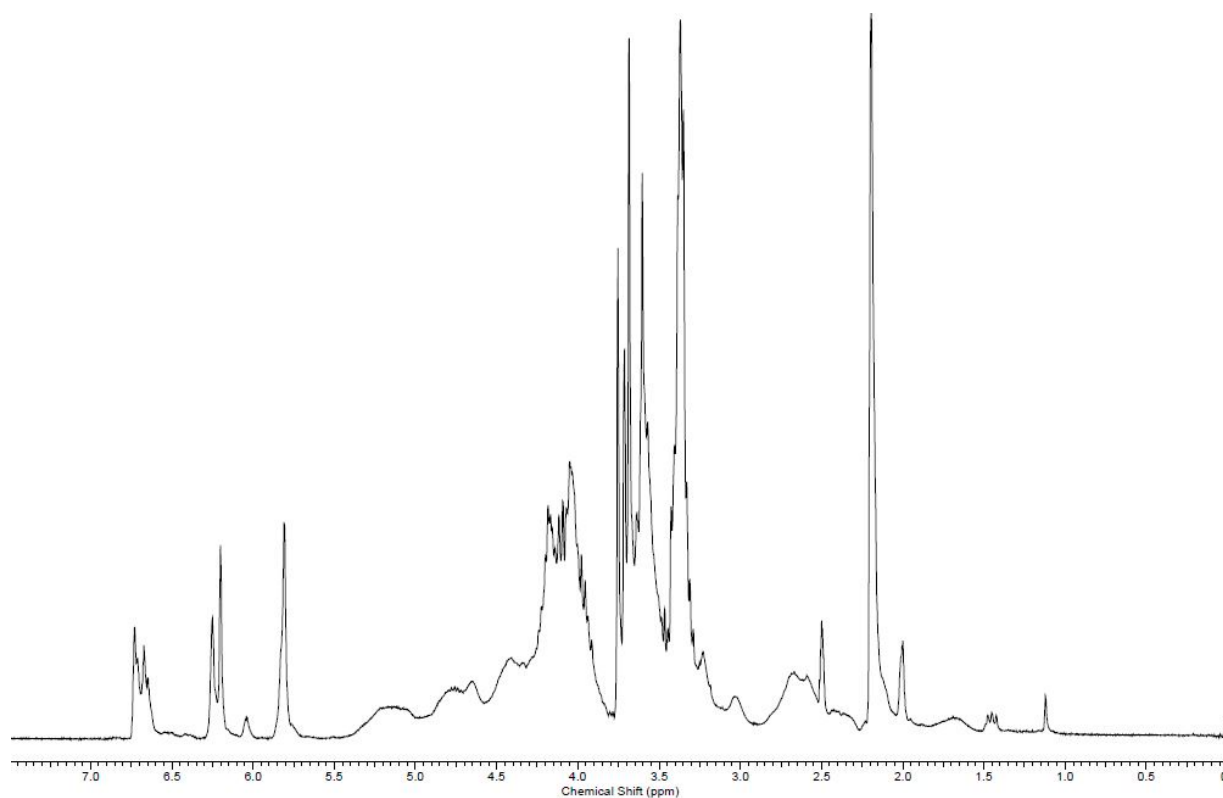

**Figure S1.**  $^1\text{H}$  NMR of PGI.

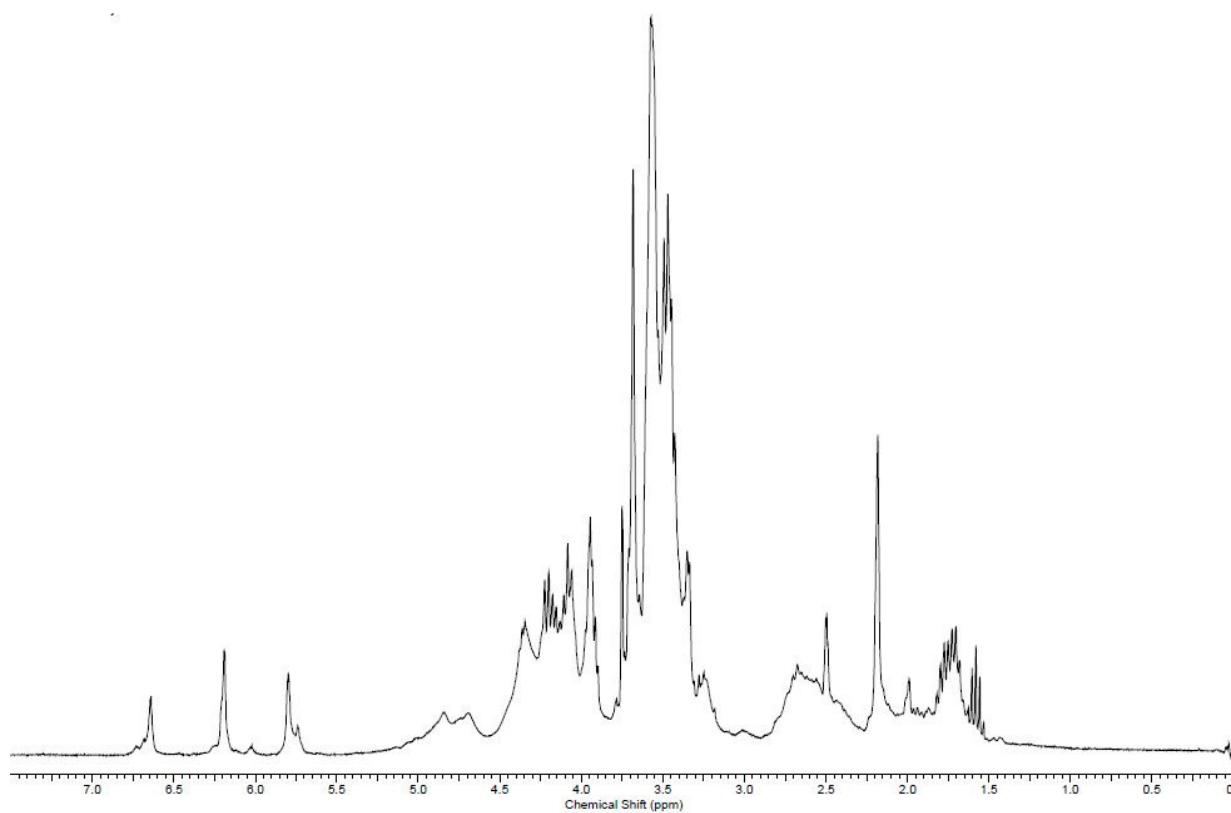

**Figure S2.**  $^1\text{H}$  NMR of PPISI.

Supplement: Supplementary file 1 [file ijms-16-14912-s001.pdf]
